# Neutrophil hitchhiking liposomal drugs for starvation therapy in endometriosis

**DOI:** 10.7150/thno.107758

**Published:** 2025-03-31

**Authors:** Mengni Zhou, Ying Wang, Caihua Li, Jinfu Li, Renbin Dou, Huanhuan Jiang, Yunjiao Zhang, Dawei Chen, Jiqian Zhang, Shasha Zhu

**Affiliations:** 1 Department of Obstetrics and Gynecology, the First Affiliated Hospital of Anhui Medical University, No 218 Jixi Road, Hefei 230022, Anhui, China.; 2 NHC Key Laboratory of Study on Abnormal Gametes and Reproductive Tract (Anhui Medical University), No 81 Meishan Road, Hefei 230032, Anhui, China.; 3 Engineering Research Center of Biopreservation and Artificial Organs, Ministry of Education, No 81 Meishan Road, Hefei 230032, Anhui, China.; 4 Department of Anesthesiology, the First Affiliated Hospital of Anhui Medical University, Key Laboratory of Anesthesiology and Perioperative Medicine of Anhui Higher Education Institutes, Anhui Medical University, No 218 Jixi Road, Hefei 230022, Anhui, China.; 5 School of Biomedical Sciences and Engineering, South China University of Technology, Guangzhou International Campus, Guangzhou 510006, China.

## Abstract

**Rationale:** Endometriosis, characterized by the presence of ectopic endometrial-like tissue, is a common chronic inflammatory disorder in gynecology. However, current treatments, including surgery and hormone therapy, often yield suboptimal outcomes and significant adverse effects. The lack of a drug delivery system specifically targeting ectopic lesions remains a major barrier to the development of more effective treatments.

**Methods:** To exploit the continuous recruitment of neutrophils and elevated reactive oxygen species (ROS) levels within endometriotic lesions, we designed a ROS-responsive liposome (cLipo) modified with neutrophil-targeting peptides on its surface. A minimally invasive mouse model of endometriosis was used to assess the neutrophil-mediated delivery of cLipo. Additionally, we co-loaded the glucose analog 2-deoxy-D-glucose (2-DG) and the autophagy inhibitor chloroquine (CQ) into the liposomes, resulting in the formulation termed cLipo-DC. The therapeutic efficacy of cLipo-DC was evaluated using human endometriotic cells (12Z), endometrial cancer cells (Ishikawa), and an endometriosis mouse model.

**Results:** Although neutrophil hitchhiking strategies are rarely reported in chronic inflammatory diseases, we found that cLipo specifically bound to neutrophils and accumulated in ectopic lesions following intraperitoneal injection in the endometriosis mouse model. Subsequently, in vitro experiments showed that cLipo-DC effectively inhibited glycolysis and autophagy in both 12Z and Ishikawa cells, resulting in cell death. Furthermore, in vivo administration of cLipo-DC in the endometriosis mouse model exerted a significant anti-endometriosis effect, with no detectable side effects.

**Conclusions:** This study provides a novel neutrophil hitchhiking platform for non-hormonal treatment of endometriosis.

## Introduction

Endometriosis, a prevalent gynecological inflammatory disorder, is characterized by the presence of endometrial-like tissue outside the uterus. It affects about 10% of women of reproductive age, frequently leading to chronic pain and infertility 1, 2. Presently, surgical and hormonal interventions represent the mainstream treatments for endometriosis. However, surgical interventions exhibit recurrence rates of up to 21.5% at 2 years and 40-50% at 5 years, while hormonal therapies pose challenges such as mood swings, pseudo-menopausal symptoms, bone density reduction, and increased risk of osteoporosis 3. Consequently, there is an urgent need for the development of more effective and tolerable treatment strategies. Recently, several nanotechnology-based therapies for endometriosis, including photothermal therapy, gene therapy, and hormone therapy, have been investigated in preclinical studies, all of which have shown promising efficacy 4-8. However, treatment approaches that are minimally invasive, have low side effects, and are non-hormonal still require further refinement.

A deeper understanding of the characteristics of endometriotic lesions could provide valuable insights for the development of targeted therapies. Since endometriosis is a chronic inflammatory disease, the immune system plays a central role in the etiology and pathophysiology of endometriosis 9-11. Neutrophils, as a crucial component of the innate immune system, have garnered increasing attention in endometriosis research. Studies have shown increased neutrophil infiltration in both systemic circulation and peritoneal fluid of endometriosis patients compared to disease-free women 12-15. Additionally, systemic circulating neutrophils from endometriosis patients exhibit a distinct transcriptomic profile compared to those from healthy controls 13. Our recent research observed persistent neutrophil enrichment in both human and mouse ectopic lesions. Conversely, although macrophages initially show enrichment towards lesions in early disease stages, this trend diminishes over time 16. These evidences underscore the potential significance of neutrophils in facilitating targeted drug delivery to lesions.

In recent years, neutrophil-based drug delivery strategies have shown tremendous potential across various diseases 17-20. These strategies mainly involve constructing neutrophil membrane-modified platforms 21-24 and neutrophil hitchhiking platforms 25-27. However, the former encounters limitations due to challenges and costs associated with large-scale production of neutrophil membranes. Neutrophil hitchhiking strategies typically entail specific binding of drugs or nanodrugs to surface molecules on neutrophils, such as CD11b, TLRs, Fcγ receptors, and formyl peptide receptors (FPRs), followed by drug delivery to lesions or target tissues by neutrophils 25, 28-32. Of note, neutrophil hitchhiking strategies are extensively employed in acute inflammation-related disorders, whereas their application in chronic inflammatory conditions such as endometriosis remains limited. Therefore, it is imperative to explore targeted drug delivery in endometriosis through the neutrophil hitchhiking strategy.

In addition to delving into specific drug delivery approaches, it is imperative to explore therapeutic agents for endometriosis that are non-hormonal and entail minimal side effects. Several studies propose caloric restriction mimetics or agents that block the energy supply to cells, showing favorable therapeutic effects in cancer treatment 33-36. In the context of endometriosis, extensive metabolic reprogramming, including aerobic glycolysis, excessive glucose intake, and high lactate production, resembling tumorigenesis, has been observed 37-39. Our previous findings consistently demonstrate a heightened demand for glucose in ectopic stromal cells 16, suggesting the potential efficacy of a glycolysis-blocking agent in treating endometriosis. Considering the metabolic reprogramming and enhanced glycolysis in endometriosis, targeting glycolysis presents a promising therapeutic strategy. Additionally, autophagy, a cellular process crucial for energy homeostasis, may support the survival of ectopic endometrial cells under metabolic stress. Therefore, we propose a combined approach of glycolysis inhibition and autophagy blockage to enhance therapeutic efficacy in endometriosis.

**Scheme 1:**
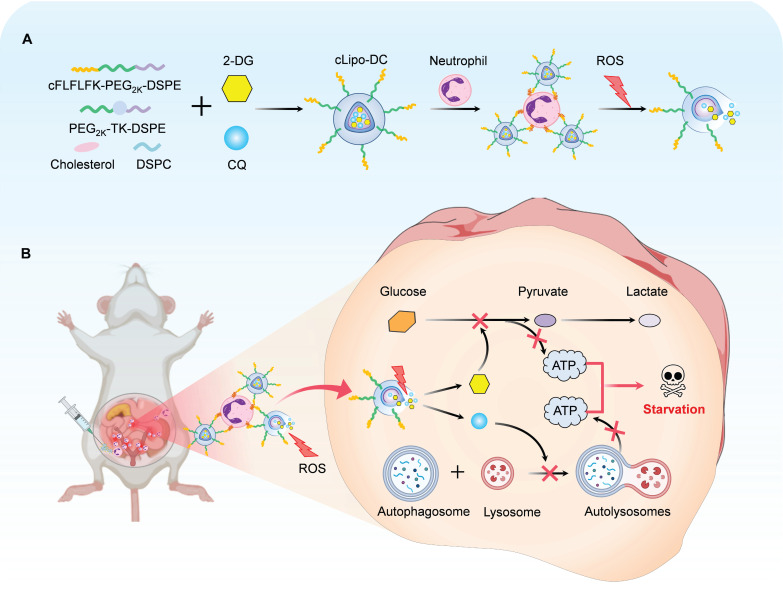
** Schematic diagram of preparation of neutrophil-hitchhiking and ROS-responsive liposome and its anti-endometriosis mechanism in mice.** (A) Liposomes were synthesized using cholesterol, DSPC, ROS-responsive polymeric DSPE-TK-PEG_2000_, and DSPE-PEG_2000_-cFLFLF, with cFLFLF peptides facilitating specific binding to neutrophils through interaction with formyl peptide receptors (FPRs). 2-DG and CQ were co-loaded into liposomes to form cLipo-DC. (B) After intraperitoneal injection, cLipo-DC undergoes specific enrichment by hitching neutrophil migration towards endometriotic lesions characterized by high ROS expression. The sustained release of 2-DG and CQ from cLipo-DC concurrently inhibits glycolysis and autophagy, thereby exerting a beneficial starvation therapy effect against endometriosis in mice.

Leveraging the continuous recruitment of neutrophils and elevated levels of reactive oxygen species (ROS) within endometriotic lesions, we designed a ROS-responsive liposome modified with neutrophil-targeting peptides on its surface. The peptide cinnamoyl-F-(D)L-F-(D)L-F (cFLFLF) is a synthetic formyl peptide receptor (FPR) antagonist that mimics the natural ligand of neutrophil FPR1, allowing it to target and bind to neutrophils with high specificity 28, 40. Furthermore, we co-loaded the glucose analog, 2-Deoxy-D-glucose (2-DG), and the autophagy inhibitor, chloroquine (CQ), into the liposomes, resulting in the formulation termed cLipo-DC. Moreover, cLipo-DC was demonstrated to simultaneously inhibit both glycolysis and autophagy, resulting in the killing of human endometriotic cells (12Z) and endometrial cancer cells (Ishikawa). Additionally, this liposome was found to bind to neutrophils and selectively accumulate in ectopic lesions upon intraperitoneal injection in a mouse model of endometriosis. Furthermore, cLipo-DC administration exerted a favorable anti-endometriosis effect with undetectable side effects. This study provides a neutrophil hitchhiking platform for non-hormonal endometriosis treatment (Scheme 1).

## Results and Discussions

### Characterization of cLipo-DC

**Figure 1:**
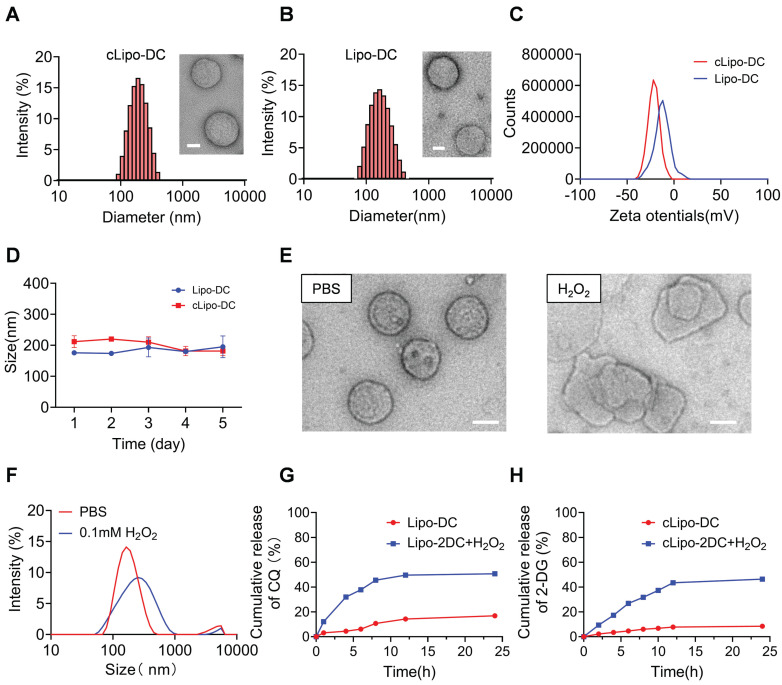
** Characterization of cLipo-DC.** (A) TEM images of cLipo-DC and its size distribution. Scale bar: 50 nm. (B) TEM images of Lipo-DC and its size distribution. Scale bar: 50 nm. (C) Zeta potential of cLipo-DC and Lipo-DC. (D) The size changes of cLipo-DC and Lipo-DC over a 5-day period. (E) TEM images of cLipo-DC and (F) its size changes upon incubation at PBS or H_2_O_2_ for 48 h. Scale bar: 100 nm. (G-H) In vitro (G) CQ and (H) 2-DG release profiles at PBS with or without H_2_O_2_.

We developed a neutrophil-hitchhiking liposome comprised of cholesterol, DSPC, ROS-responsive polymeric DSPE-TK-PEG_2000_, and DSPE-PEG_2000_-cFLFLF, with cFLFLF peptides facilitating specific binding to neutrophils by interacting with FPRs (Figure S1) 28, 40. The glycolysis inhibitor 2-DG and the autophagy blocker CQ were encapsulated into liposomes using the passive method and the active transmembrane ammonium sulfate gradient method, respectively 41, 42. This liposome formulation is referred to as cLipo-DC, while the liposome without targeted peptide modification was named Lipo-DC. The loading content (LC) of 2-DG and CQ was determined to be 18.45 ± 0.48% and 5.04 ± 0.38%, respectively; while the encapsulation efficiency (EE) was 48.39 ± 1.26% and 35.31 ± 2.65%, respectively (Table S1). cLipo-DC and Lipo-DC exhibited a uniform spherical shape with average sizes of 201.8 ± 7.81 nm and 175.32 ± 2.63 nm, respectively (Figure 1A-B). Both cLipo-DC and Lipo-DC exhibited a negative zeta potential of -21.51 mV and -12.47 mV, respectively (Figure 1C). The polydispersity index (PDI) in phosphate-buffered saline (PBS) was 0.16 for cLipo-DC and 0.13 for Lipo-DC, indicating good aqueous dispersity. Additionally, both formulations remained stable in PBS for at least 5 days (Figure 1D). Observations under transmission electron microscopy (TEM) revealed that the spherical morphology of the liposomes disappeared upon exposure to H_2_O_2_ (Figure 1E). Additionally, dynamic light scattering (DLS) analysis showed an obvious increase in the average particle size of the liposomes after incubation with H_2_O_2_ (Figure 1F). These results demonstrated the good ROS responsiveness of cLipo-DC. Moreover, the cumulative release of 2-DG and CQ from cLipo-DC liposome was determined by analyzing the released medium. As shown in Figure 1G-H, sustained and slow drug release was observed without H_2_O_2_, while increased drug release behavior was observed under an H_2_O_2_ atmosphere. Release of CQ and 2-DG began within 30 min and reached a maximum at 12 h (Figure 1G-H). Notably, the non-ROS-responsive cLipo-DC (NRcLipo-DC), which did not contain DSPE-TK-PEG_2000_, did not exhibit H₂O₂-induced drug release capability (Figure S2). These results indicate that cLipo-DC possesses a favorable ability to slowly release drugs in response to ROS.

#### Evaluation of the cytotoxicity of cLipo-DC in vitro

**Figure 2:**
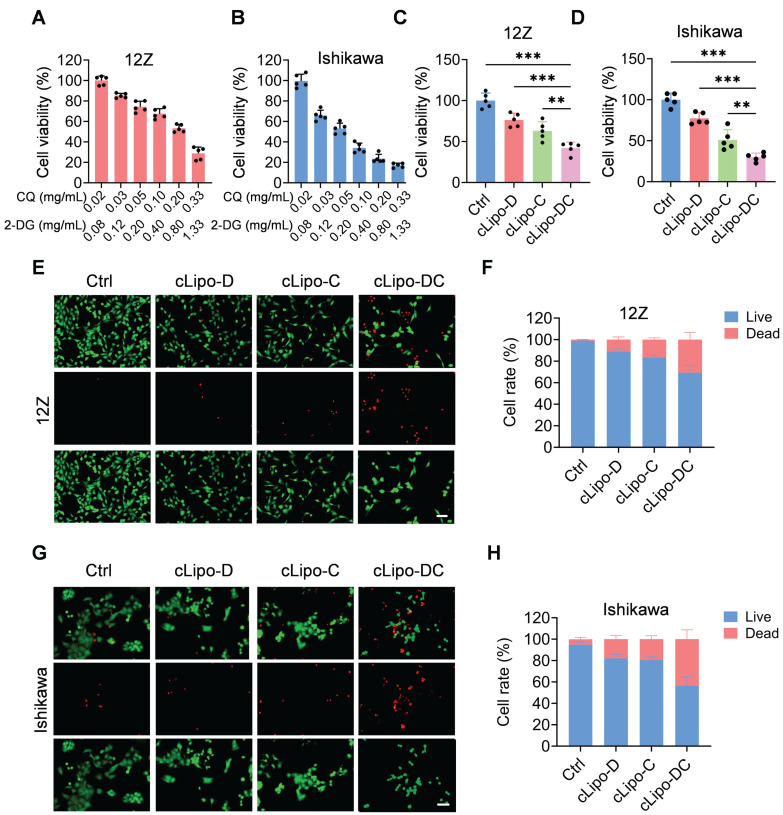
** Cytotoxicity of cLipo-DC in vitro.** (A-B) Relative cell viability of (A) 12Z and (B) Ishikawa cells treated with various concentrations of cLipo-DC for 48 h (C-D) Relative cell viability of (C) 12Z and (D) Ishikawa cells treated with different formations for 48 h. (E) Live/dead staining images and corresponding statistical results of (E-F) 12Z and (G-H) Ishikawa cells stained with calcein-AM/ propidium iodide after incubation with various formulations for 48 h. Scale bar is 50 μm. Data are shown as the mean ± SD and analyzed by one-way ANOVA with Turkey's post hoc test. **P < 0.01, and ***P < 0.001.

We subsequently assessed the cytotoxicity of cLipo-DC on 12Z and Ishikawa cell lines. As shown in Figure 2A-B, upon 48 h of incubation, demonstrated a concentration-dependent reduction in cell viability for both 12Z and Ishikawa cells. Then, we proceeded to validate the efficacy of each component within the cLipo-DC. As shown in Figure 2C, compared to cells treated with cLipo-D or cLipo-C, cLipo-DC treatment demonstrated significant lower cell viability. Furthermore, the cells treated with cLipo-DC exhibited the most pronounced decrease in cell viability among 12Z cells. In addition, in Ishikawa cells, cLipo-DC demonstrated comparable cytotoxic effects (Figure 2D). As shown in Figure 2E-H, cells treated with cLipo-DC exhibited a more significant decrease in viable cells and an increase in dead cells compared to the cLipo-D, cLipo-C, and control treatments. These findings underscore the potent cell-killing efficacy of the combination therapy involving 2-DG and CQ.

### Validation of glycolysis inhibition and autophagy impairment by cLipo-DC

**Figure 3:**
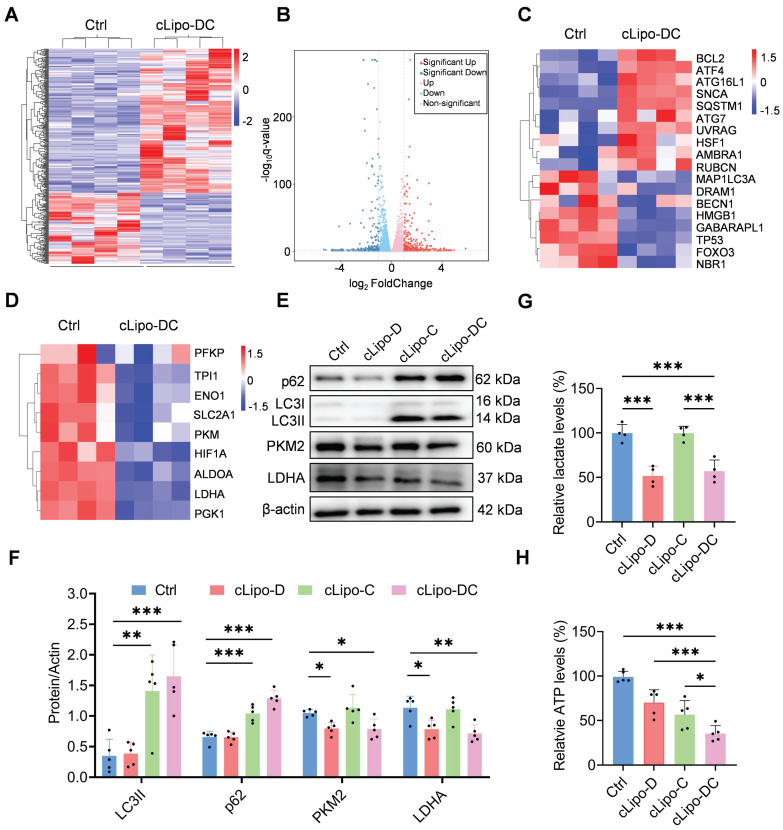
** cLipo-DC inhibits glycolysis and autophagy in vitro.** (A) Heatmap and (B) Volcano plots depicting genes differentially expressed between control and cLipo-DC treated 12Z cells. (C) Heatmaps showing expression of (C) autophagy-associated genes and glycolysis-associated genes (D) in control and cLipo-DC treated 12Z cells. (E-F) Western blotting analyses of p62, LC3, PKM2, and LDHA expression levels in 12Z cells after different treatments for 48 h. β-actin expression levels serve as the loading controls. (G) Lactic acid and (H) ATP contents of 12Z cells after different treatments for 48 h. Data are shown as the mean ± SD and analyzed by one-way ANOVA with Turkey's post hoc test. *P < 0.05, **P < 0.01, and ***P < 0.001.

To elucidate the cell-killing mechanism of cLipo-DC, we analyzed the differential transcriptome of 12Z cells by using RNA sequencing. Compared with the control group, 268 genes were upregulated and 176 genes were downregulated in the cLipo-DC treated cells (Figure 3A-B). Notably, cLipo-DC treatment led to significant changes in genes associated with autophagy and glycolysis (Figure 3C-D), suggesting the impairment of autophagy and inhibition of glycolysis. Next, the different expression of key genes associated with autophagy and glycolysis were validated by western blotting. LC3 serves as a marker protein for autophagy, whereas p62 acts as a substrate protein in the autophagic process. 43, 44. We found that the levels of LC3 II and p62 proteins significantly increased in cells treated with CQ-containing formulations (cLipo-C and cLipo-DC) compared to the control group (Figure 3E-F), indicating the inhibition of the autophagic degradation process 45. Additionally, PKM2 and LDHA are key enzymes in glycolysis. PKM2 catalyzes the conversion of phosphoenolpyruvate to pyruvate, while LDHA converts pyruvate to lactate, supporting energy production 46, 47. As shown in Figure 3E-F, cells treated with 2-DG containing formulations (cLipo-D and cLipo-DC) exhibited a significant decrease in PKM2 and LDHA levels compared to control cells. Furthermore, the levels of lactate, an important product of glycolysis, significantly decreased in cells treated with 2-DG containing formulations compared to the control group, further confirming the inhibition of glycolysis (Figure 3G). Finally, the impact of cLipo-DC treatment on inhibiting ATP generation was significantly more pronounced than the additive effect of its individual components (Figure 3H), indicating a synergistic effect among these components in inducing energy deprivation in vitro.

### Highly specific accumulation of cLipo in ectopic lesions

**Figure 4:**
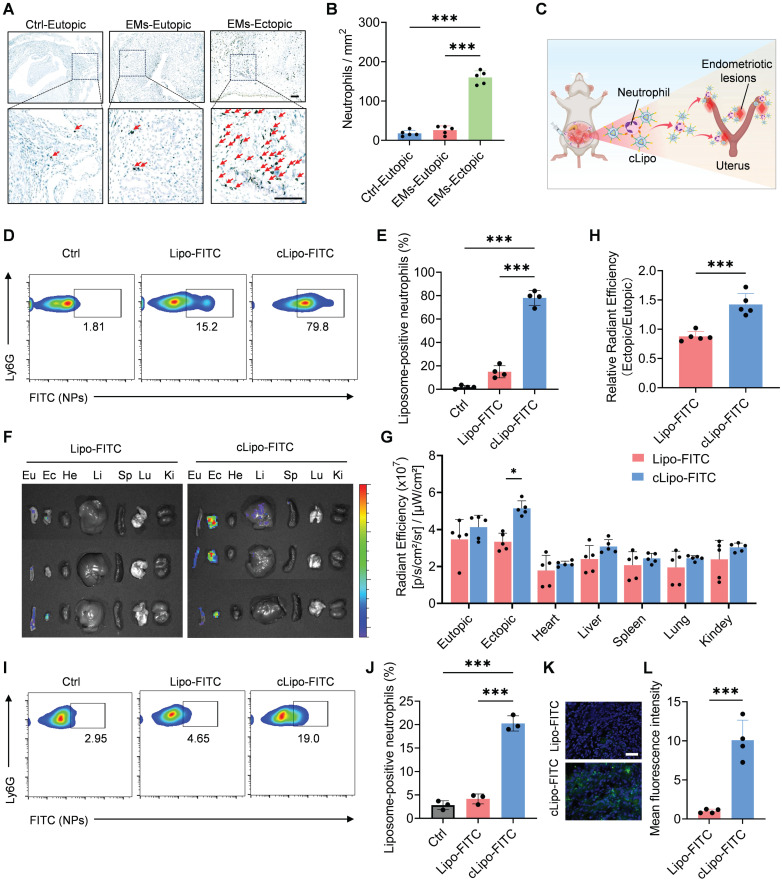
** Highly specific accumulation of cLipo in ectopic lesions.** (A) Representative Ly6G immunohistochemistry of ectopic ovarian endometriosis (EMs-Ectopic), matched eutopic endometrium (EMs-Eutopic), and control endometrium (Ctrl-Eutopic) in mouse. The scale bar is 100 μm. (B) The number of Ly6G ^+^ neutrophils per field in ectopic ovarian endometriosis, matched eutopic endometrium, and control endometrium. N = 5 per group. (C) Schematic diagram showing neutrophil-mediated delivery of liposomes in ectopic lesions. (D-E) Flow cytometry analysis of the percentage of liposome-positive neutrophils (FITC^+^CD11b^+^Ly6G^+^) in peritoneal fluid 2 h after intraperitoneal injection. N = 4 per group. (F) Ex vivo fluorescence images and (G) corresponding quantification of average fluorescence intensities of eutopic endometrium, ectopic lesion and major organs collected 16 h post liposomes injection by intraperitoneal. N = 5 per group. (H) Ratio of ectopic/eutopic fluorescence in (F). N = 5 per group. (I-J) Flow cytometry analysis of the percentage of liposome-positive neutrophils (FITC^+^CD11b^+^Ly6G^+^) in ectopic lesions 16 h after intraperitoneal injection. N = 3 per group. (K) Fluorescent images of frozen sections of ectopic lesions and (L) their statistical results. Data are shown as mean ± SD and analyzed by one-way ANOVA with Turkey's post hoc test or two-tailed t-test. *P < 0.05, ***P < 0.001.

Before verifying the in vivo efficacy of liposomal drugs for anti-endometriosis treatment, we investigated their targeting to ectopic lesions and biodistribution. As shown in Figure 4A-B, neutrophils were significantly increased in ectopic endometriosis compared with either matched eutopic endometrium or control endometrium in the mouse model. These findings suggest that neutrophils may serve as targets for delivering cLipo to ectopic lesions (Figure 4C). To test this hypothesis, fluorescent dye FITC was employed to simulate loaded drugs and encapsulated within liposomes modified with neutrophil-targeting peptides (cLipo-FITC), as well as within unmodified liposomes (Lipo-FITC). To confirm that cLipo can hitchhike on neutrophils, we initially co-incubated liposomes with neutrophils in vitro. We observed that cLipo-FITC exhibited higher efficiency in hitchhiking on neutrophils compared to Lipo-FITC (Figure S3). Subsequently, we intraperitoneally injected liposomes into mice and used flow cytometry to detect liposomes hitchhiking on neutrophils. The results demonstrated that, compared to the mice treated with Lipo-FITC or the control group, those treated with cLipo-FITC exhibited a significant increase in both the percentage of liposome-positive neutrophils and the mean fluorescence intensity of FITC-loaded liposomes within the neutrophils. (Figure 4D-E, Figure S4-5). These results highlight that cLipo effectively hitchhike on neutrophils.

Next, the distribution of liposomes in vivo was assessed using a fluorescence imaging system. As shown in Figure 4F-G, the highest accumulation of cLipo-FITC in ectopic lesions was observed after intraperitoneal injection, whereas the accumulation of Lipo-FITC in ectopic lesions after injection was significantly lower than that of cLipo-FITC. The ratio of fluorescence in ectopic lesions versus eutopic endometrium also reflected a specifically higher distribution in ectopic lesions following intraperitoneal injection (Figure 4H). Additionally, ectopic lesion tissues were harvested and analyzed by flow cytometry, revealing that 19.00% of neutrophils were cLipo-FITC-positive, significantly higher than the 4.65% observed for Lipo-FITC (Figure 4I-J). Furthermore, tissue section analysis demonstrated that fluorescence intensity in ectopic tissues was markedly higher in mice treated with cLipo-FITC compared to those receiving Lipo-FITC (Figure 4K-L). Taken together, these results demonstrate that intraperitoneally injected cLipo is a highly specific delivery platform for endometriotic lesions.

To verify neutrophil-mediated delivery, we treated mice with a neutralizing antibody against neutrophils (anti-Ly6G) and a CXCR2/CXCR1 antagonist, then evaluated cLipo delivery. The results showed that both treatments significantly reduced cLipo accumulation at the lesion site (Figure S6). These findings indicate that cLipo is directly transported to the lesion site by peritoneal neutrophils through a CXCR1/2-mediated recruitment mechanism. Additionally, immunofluorescence staining of the lesion showed that, after neutrophil depletion, both cLipo-FITC and Ly-6G-labeled neutrophil signals were reduced at the lesion, and their co-localization was no longer observed (Figure S7). Additionally, we observed that as early as 8 h after intraperitoneal injection, neutrophils had delivered cLipo to the ectopic lesions. During this period, cLipo-DC had no significant impact on neutrophil viability, mobility, or immunogenicity (Figure S8).

### In vivo anti‑endometriosis performance of cLipo-DC

**Figure 5:**
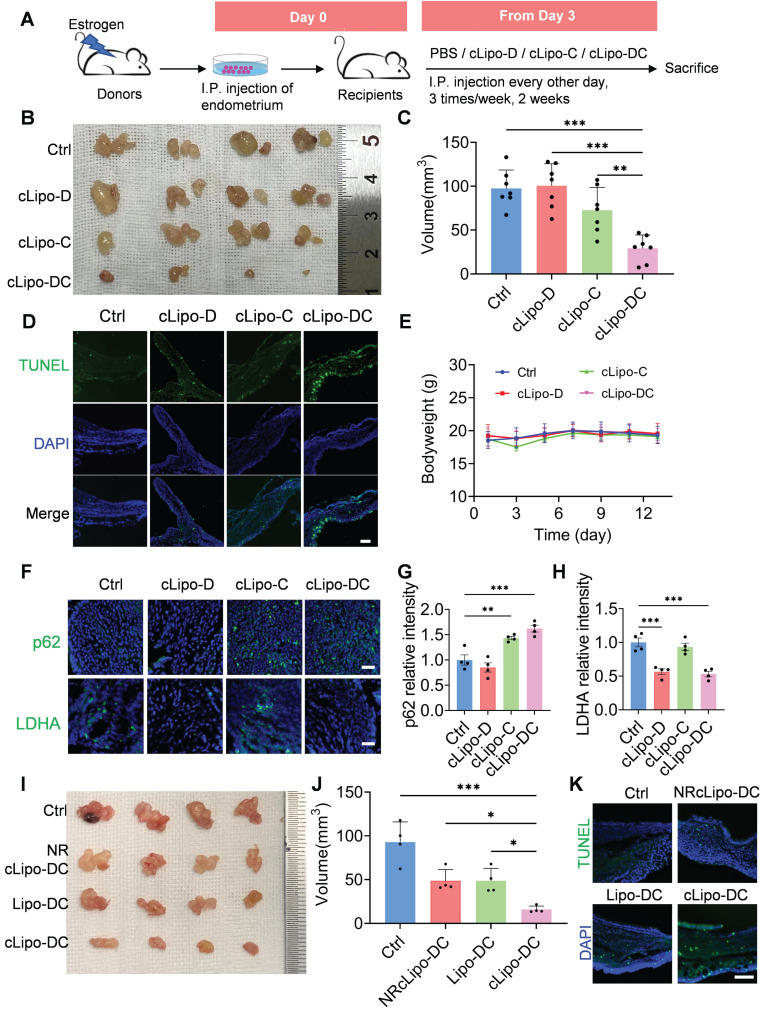
** Anti-endometriosis performance of cLipo-DC.** (A) Schematic drawing of treatment with different formations, starting from the Day 3. (B) Images and (C) total volume of endometriotic lesions in mice following different treatments. N = 7 per group. (D) Fluorescence images of TUNEL staining in endometriotic lesions in mice following different treatments. Blue staining is DAPI, green staining is TUNEL. Scale bar is 100 μm. (E) Monitoring the body weight of mice receiving different treatments. (F) Immunofluorescence staining of p62 and LDHA in endometriotic lesions in mice following different treatments, along with (G-H) their statistical analysis of relative fluorescence intensity. Sacle bar is 50 μm. (I) Images and (J) total volume of endometriotic lesions in mice following different treatments. N = 4 per group. (K) Fluorescence images of TUNEL staining in endometriotic lesions in mice following different treatments. Scale bar is 100 μm. Data are shown as mean ± SD and analyzed by one-way ANOVA with Turkey's post hoc test or two-tailed t-test. *P < 0.05, **P < 0.01, ***P < 0.001.

To assess the effectiveness of cLipo-DC in treating endometriosis, we initially established a minimally invasive mouse model of endometriosis by transplanting minced endometrial fragments from donor mice into the peritoneum of syngeneic recipient mice, following established protocols 48, 49 (Figure 5A). Consistent with prior findings 50, 51, we observed a notable increase in ROS levels at endometrial lesion sites (Figure S9), suggesting the potential for cLipo-DC to release drugs responsively at these sites. Subsequently, recipient mice were randomly divided into four groups to evaluate the treatment efficacy of different formations (Figure 5A). Following intraperitoneal injection of cLipo-DC, an obvious reduction in the size of endometriotic lesions in mice was observed (Figure 5B). Statistical analysis revealed that the lesion volumes in cLipo-DC-treated mice were significantly smaller than those in control, cLipo-D, and cLipo-C-treated mice (Figure 5C). Analysis of apoptosis via the terminal deoxynucleotidyl transferase dUTP nick end labeling (TUNEL) assay revealed increased green fluorescence signals in cLipo-DC-treated mice, indicative of a higher level of apoptotic cells (Figure 5D). Importantly, throughout the treatment period, no significant changes in the body weight of the mice were noted in any of the groups (Figure 5E). Furthermore, immunofluorescence staining of lesion sections showed elevated p62 levels in mouse lesions following cLipo-C and cLipo-DC treatments, while LDHA levels decreased after cLipo-D and cLipo-DC treatments (Figure 5F-H). This suggests that cLipo-DC could concurrently inhibit glycolysis and block autophagy within the lesions. Finally, we evaluated the impact of ROS responsiveness and neutrophil hitchhiking on the anti-endometriosis efficacy of cLipo-DC. As shown in Figure 5I-J, mice treated with NRcLipo-DC or Lipo-DC exhibited significantly larger ectopic lesions and fewer apoptotic cells compared to those treated with cLipo-DC. Moreover, in mice receiving a neutrophil-neutralizing antibody, the therapeutic efficacy of cLipo-DC was markedly reduced (Figure S10). These findings highlight the essential role of ROS responsiveness and neutrophil hitchhiking in the potent anti-endometriosis effects of cLipo-DC.

Notably, the results of the hemolysis assay and H&E staining indicated that cLipo-DC treatments did not induce red blood cell lysis or cause significant changes in the function of major organs, underscoring its favorable biosafety profile (Figure S11-12).

## Materials and methods

### Materials

Cholesterol (57-88-5) and (NH_4_)_2_SO_4_ (7783-20-2) were purchased from Sigma-Aldrich. Fluorescein 5(6)-isothiocyanate (F809567) was purchased from Macklin. 2-Deoxy-D-glucose (HY-13966) and Chloroquine (HY-17589) were purchased from MCE. DSPE-PEG_2000_-cFLFLF (R-DT-0623), DSPE-TK-PEG_2000_ (R-D526) and DEPC (LP-R4-076) were purchased from Xi'an ruixi Biological Technology Co., Ltd. Cell Counting Kit-8 (E-CK-A362) and L-Lactic Acid (LA) Colorimetric Assay Kit (E-BC-K044-M) were purchased from Elabscience. Anti-CD16/32 (101319), anti-CD11b (101228), anti-Ly6G (127607) were purchased from BioLegend. Recombinant Anti-SQSTM1/p62 antibody (EPR4844) and recombinant Anti-β-Actin antibody (ab8226) were purchased from Abcam. LC3 Polyclonal antibody (14600-1-AP), LDHA-Specific Polyclonal antibody (19987-1-AP), and PKM2-specific Polyclonal antibody (15822-1-AP) were purchased from Proteintech. Super-sensitive ECL chemiluminescent substrate (BL520A) was purchased from Biosharp. ATP Content Assay Kit (BC0300) was purchased from Solarbio. Calcein AM and Propidium Iodide (PI) (C2015S), Dihydroethidium (S0063) and One Step TUNEL Apoptosis Assay Kit (Green Fluorescence) (C1088) were purchased from Beyotime. Ly6G-specific polyclonal antibody (GB11229-100) were purchased from Servicebio. Sephadex G-50 Medium (17004301) was purchased from Cytiva.

### Synthesis and characterization of liposomes

DSPE-PEG_2000_-cFLFLF was synthesized by Xi'an ruixi Biological Technology Co. Synthetic route is as follows: DSPE-PEG_2000_-NHS (100 mg) was dissolved in 3 mL of DMF. Peptide (1.1 eq.) and triethylamine (3.0 eq.) were added and allowed to react completely at room temperature for 12 h. The reaction mixture was transferred to a dialysis bag (molecular weight cutoff 2500 Da) and dialyzed against pure water for 24 h. The dialysate was collected and freeze-dried to yield the desired product.

The liposomes were prepared as follows: 60 mg of DSPC (LP-R4-076, Xi'an ruixi Biological Technology Co.), 10 mg of DSPE-TK-PEG_2000_ (R-D526, Xi'an ruixi Biological Technology Co.), 10 mg of DSPE-PEG_2000_-cFLFLF (R-DT-0623, Xi'an ruixi Biological Technology Co.), and 20 mg of cholesterol (57-88-5, Sigma-Aldrich) were dissolved in 400 μL of anhydrous ethanol. The mixture was heated at 60 °C for 30 min to ensure complete lipid dissolution, yielding an alcohol-phase solution. While stirring continuously, the alcohol-phase solution was slowly added dropwise to a water-phase solution maintained at 60 °C, containing 2 mL of 40 mg/mL 2-DG (HY-13966, MCE) and 250 mM (NH_4_)_2_SO_4_ (7783-20-2, Sigma-Aldrich), to produce liposomes. The mixture was stirred at 1200 rpm for 1 h to ensure thorough hydration. Subsequently, the liposomes were extruded through a 100 nm polycarbonate membrane to attain the desired particle size. The product was dialyzed in a dialysis bag (molecular weight cutoff: 8-10 kDa) for 12 h to remove unreacted 2-DG. After dialysis, the liposomes were combined with 2 mL of a 15 mg/mL CQ (HY-17589, MCE) solution pre-warmed to 40 °C and the mixture was heated at 40 °C for 10-15 min. The free drug was then eliminated using a G50 Sephadex gel column (17004301, Cytiva). The morphology of cLipo-DC was characterized by TEM (JEOL-2010). Additionally, cLipo-DC was characterized by DLS using a particle size analyzer (90 Plus, Brookhaven Instruments Co.). The particle size distribution and zeta potential of cLipo-DC were determined using DLS and a particle size analyzer (90 Plus, Brookhaven Instruments Co.).

The content of 2-DG was determined using a Sciex Triple Quad 4500 high-performance liquid chromatography (HPLC) system equipped with a C18 column (Thermo HYPERSIL GOLD C18). The content of CQ was determined using a Micro UV-Vis Spectrophotometer (LIFEREAL, FC-1100) at an absorbance wavelength of 343 nm.

The synthesis of cLipo-FITC was similar to the above-mentioned, except that the loaded drugs was changed to 2 mL of 0.5 mg/mL FITC aqueous solution. Compared with the synthesis of cLipo-FITC, the synthesis of Lipo-FITC involved replacing DSPE-PEG_2000_-cFLFLF with 10 mg of DSPE-PEG_2000_ (R-1028-2K, Xi'an ruixi Biological Technology Co.).

### ROS-responsive drug release

mL of cLipo-DC was placed into a dialysis bag with a molecular weight cutoff of 8-10 kDa and immersed in 30 mL of PBS solution (with/without 100 μM H2O2) at 37 °C, with shaking at 100 rpm in a water bath. At indicated time points, a 100 μL PBS sample from outside the dialysis bag was collected and replaced with an equal volume of fresh PBS (with/without 100 μM H2O2). Morphological changes in the liposomes, with/without 100 μM H2O2, were observed using TEM (JEOL-2010). Changes in liposome particle size at different time points were measured using DLS. The amounts of 2-DG and CQ released from cLipo-DC were quantified using a Sciex Triple Quad 4500 HPLC system and a Micro UV-Vis Spectrophotometer (LIFEREAL, FC-1100), respectively.

### Cell viability assay

The Ishikawa cell line was obtained from the Cell Bank of the Type Culture Collection of the Chinese Academy of Sciences, and the 12Z cell line was purchased from Zhejiang Meisen Cell Technology Co., Ltd. Cells were seeded into 96-well plates (12Z: 1×10^4^ cells/ well, Ishikawa: 2×10^4^ cells/ well) and cultured overnight at 37 °C with 5% CO_2_. The next day, the cells were treated for 48 h with either various concentrations of cLipo-DC, or fixed concentrations of cLipo-DC (2-DG: 0.80 mg/mL, CQ: 0.20 mg/mL), cLipo-D (2-DG: 0.80 mg/mL), cLipo-C (CQ: 0.20 mg/mL). Next, 10 μL of Cell Counting Kit-8 (E-CK-A362, Elabscience) was added to each well and incubated at 37 °C for 30 min. The absorbance was measured at 450 nm using a microplate reader (Nano Quant, Tecan).

### Staining of live/dead cells

Cells treated with fixed concentrations of cLipo-D (2-DG: 0.80 mg/mL), cLipo-C (CQ: 0.20 mg/mL), cLipo-DC (2-DG: 0.80 mg/mL, CQ: 0.20 mg/mL) were washed and stained with Calcein AM and Propidium Iodide (PI) (C2015S, Beyotime). And then incubated at 37 °C in the dark for 30 min. Stained cells were then imaged using fluorescence microscopy.

### Western blotting

After various treatment, 12Z Cells were harvested and lysed with sample buffer and boiled for 10 min. Proteins were separated by sodium dodecylsulfate polyacrylamide gel electrophoresis and were transferred to nitrocellulose membranes. The membranes were incubated with p62 (EPR4844, Abcam), LC3 (14600-1-AP, Proteintech), LDHA (19987-1-AP, Proteintech), PKM2 (15822-1-AP, Proteintech), and β-actin (ab8226, Abcam) at 4 °C overnight, then with secondary antibodies for 1 h at 37 °C. Membranes were incubated with ECL kit reagents and visualized using a chemiluminescence instrument (ImageQuant LAS 4000, GE Healthcare).

### In vitro detection of lactic acid and ATP content

12Z cells were cultured and treated with cLipo-D (2-DG: 0.80 mg/mL), cLipo-C (CQ: 0.20 mg/mL), or cLipo-DC (2-DG: 0.80 mg/mL, CQ: 0.20 mg/mL for 48 h. The culture medium was collected for detecting the secreted lactic acid content use a L-Lactic Acid (LA) Colorimetric Assay Kit (E-BC-K044-M). Furthermore, cells are collected after digestion with trypsin, followed by homogenization using a homogenizer (JXFSTPRP, Jingxin). The homogenates were then centrifuged at 4 ℃ for 10 min at 10,000 g. The supernatants were collected, and the intracellular ATP contents were measured using an ATP Content Assay Kit (BC0300, Solarbio).

### mRNA sequencing

mRNA isolation, library preparation, sequencing, and differential expression analysis were conducted by OE Biotech Co., Ltd. (Shanghai, China). 12Z cells were cultured and treated with either cLipo or cLipo-DC for 48 h. Total RNA was extracted using TRIzol reagent following the manufacturer's protocol. RNA purity and quantity were assessed using a NanoDrop 2000 spectrophotometer (Thermo Scientific), while RNA integrity was evaluated using an Agilent 2100 Bioanalyzer (Agilent Technologies, Santa Clara). Transcriptome libraries were prepared using the VAHTS Universal V6 RNA-seq Library Prep kit as per the manufacturer's instructions. The Illumina NovaSeq 6000 platform was employed to sequence the libraries, producing 150 bp paired-end reads (~50 million reads/sample). Initial processing of raw reads utilized fastp software to eliminate low-quality reads, resulting in approximately 48 million clean reads. These reads underwent mapping to the reference genome using a hierarchical index tailored for transcript splicing comparisons. Gene expression levels were quantified using exon per kilobase/million mapped fragments (FPKM), with read counts determined by HTSeq-count. Principal component analysis (PCA) was conducted in R (v3.2.0) to evaluate the biological consistency across samples. Differential gene expression (DEG) analysis was performed using DESeq2 software, identifying genes with a q-value < 0.05 and a fold change > 2 or < 0.5 as significant DEGs. Hierarchical clustering of DEGs was conducted in R (version 3.2.0) to visualize gene expression patterns across different groups and samples. Bioinformatic analysis was performed using the OECloud tools at https://cloud.oebiotech.com/task/.

### Mice

Female Balb/c mice, aged six to eight weeks, weighing 18 to 25 g, were purchased from GemPharmatech Co., Ltd. The mice were kept in controlled environments with regulated temperature, humidity, and lighting, and were provided with unrestricted access to water. All procedures involving the animals complied with the Ethical Regulations on the Care and Use of Laboratory Animals established by Anhui Medical University and received approval from the university's animal experiment committee.

### Model of endometriosis

The mouse model of endometriosis was performed as described previously 48, 49. Donor mice received subcutaneous injections of estradiol benzoate (3 μg/mouse, MCE). After one week of estrogen injections, the donor mice were euthanized, and their uterine horns were collected and longitudinally cut with scissors. Each uterine horn was meticulously dissected into fragments. Fragments from the uterine horns of two mice were intraperitoneally injected into four recipient mice to induce endometriosis.

### Flow cytometry analysis

Mice were intraperitoneally injected with 100 μL 50 mg/mL cLipo, Lipo-FTIC, or cLipo-FITC and euthanized at the indicated time. Peritoneal fluids were collected and centrifuged at 450 g for 5 min. Ectopic lesions were minced into small pieces and incubated in PBS containing 1mg/mL collagenase type IV and 40ug/mL DNase I for 45 min at 37 °C with shaking at 100 rpm, followed by centrifugation at 450 g for 5 min. Cell suspensions were incubated with anti-CD16/32 (101319, BioLegend) for Fc blocking for 10 min. Subsequently, cells were stained with PerCP/Cyanine5.5-conjugated anti-CD11b (101228, BioLegend), PE/Cyanine7-conjugated anti-CD80 (104733, BioLegend), APC-conjugated anti-CD86 (105011, BioLegend), and PE-conjugated anti-Ly6G (127607, BioLegend) monoclonal antibodies (mAbs) for 30 min on ice. Samples were acquired by a BD FACSVerse flow cytometer, and the data were analyzed with FlowJo V10 software.

### Distribution of liposomes in mice

Endometriosis mice were intraperitoneally injected with 100 μL 50 mg/mL of Lipo-FITC or cLipo-FITC. After 16 h, eutopic endometrium, ectopic lesions, and major organs including heart, liver, spleen, lungs, kidneys, were collected. Fluorescent images of these organs were acquired using an IVIS III imaging system (PerkinElmer), and the intensity of the probe signal was automatically calculated based on their spectral patterns using commercial software.

### In vivo therapy

Endometriosis mice were divided into groups and received intraperitoneal injections of 200 μL of cLipo, cLipo-D (2-DG: 8.00 mg/mL), cLipo-C (CQ: 2.00 mg/mL), and cLipo-DC (2-DG: 8.00 mg/mL, CQ: 2.00 mg/mL), every other day, starting from day 3 after injection of donor endometrial debris. Body weights were measured every other day. After 2 weeks, the mice were euthanized individually, and their lesions were excised and processed for disease assessment or immunofluorescence evaluation. Lesions were measured with calipers, and the extent of endometriosis was assessed by calculating the total volume of all lesions in each mouse. The volume of each lesion was determined using the formula: 0.5 × length × width² 16. To validate the impact of ROS responsiveness and neutrophil hitchhiking on the anti-endometriosis efficacy of cLipo-DC, the endometriosis mice were divided into four groups, each receiving treatment with cLipo, NRcLipo-DC, Lipo-DC, or cLipo-DC. The dosing regimen and assessment of anti-endometriosis effects were performed as described above.

### Assessments of TUNEL

To detect apoptosis in the injured endometriosis lesions, the frozen sections were subjected to terminal deoxynucleotidyl transferase-mediated dUTP-biotin nick end labeling (TUNEL) assay (C1088, Beyotime) following the manufacturer's instructions.

### Statistical analysis

Data are presented as mean ± SD, as indicated in the figure legends. Statistical analysis was conducted using GraphPad Prism 9.0 software, employing one-way analysis of variance (ANOVA) followed by Tukey's post hoc test or two-tailed t-test as appropriate. Differences with P < 0.05, P < 0.01, and P < 0.001 were considered statistically significant and denoted with *, **, and ***, respectively.

## Conclusion

A novel non-hormonal treatment platform for endometriosis has been developed using ROS-responsive liposomes modified with neutrophil-targeting peptides. These liposomes were shown to specifically accumulate at ectopic lesions by hitchhiking on neutrophils after intraperitoneal injection in a mouse model of endometriosis. Furthermore, co-loading 2-DG and CQ into the liposomes resulted in favorable anti-endometriosis effects through simultaneous inhibition of glycolysis and autophagy, with minimal observed side effects. This study provides a neutrophil hitchhiking platform for the drug therapy of endometriosis.

## Supplementary Material

Supplementary materials and methods, figures and table.
