# Supplementary material for: Neutrophil hitchhiking liposomal drugs for starvation therapy in endometriosis
Source: Theranostics. 2025 Mar 31;15(10):4848–60. doi: 10.7150/thno.107758 (PMC11984412; doi:10.7150/thno.107758)
Supplement: Supplementary file 1 — Supplementary materials and methods, figures and table. [file thnov15p4848s1.pdf]

Supplementary information

Neutrophil hitchhiking liposomal drugs for starvation therapy in endometriosis

Mengni Zhou [1,2,3+], Ying Wang [4+], Caihua Li [1,2,3+], Jinfu Li [4], Renbin Dou [1,2,3], Huanhuan Jiang [1,2,3], Yunjiao Zhang [5], Dawei Chen [1,2,3], Jiqian Zhang [4]*, Shasha Zhu [1,2,3]**

1. *Department of Obstetrics and Gynecology, the First Affiliated Hospital of Anhui Medical University, No 218 Jixi Road, Hefei 230022, Anhui, China*
2. *NHC Key Laboratory of Study on Abnormal Gametes and Reproductive Tract (Anhui Medical University), No 81 Meishan Road, Hefei 230032, Anhui, China*
3. *Engineering Research Center of Biopreservation and Artificial Organs, Ministry of Education, No 81 Meishan Road, Hefei 230032, Anhui, China*
4. *Department of Anesthesiology, The First Affiliated Hospital of Anhui Medical University, Key Laboratory of Anesthesiology and Perioperative Medicine of Anhui Higher Education Institutes, Anhui Medical University, Hefei, Anhui, China*
5. *School of Biomedical Sciences and Engineering, South China University of Technology, Guangzhou International Campus, Guangzhou, China.*

[+] These authors contributed equally to this work

[*] To whom correspondence should be addressed
E-mail:
chendawei0930@163.com; jiqianzh@mail.ustc.edu.cn; zss90328@mail.ustc.edu.cn

Materials and methods

Isolation of mouse neutrophils

Mice were euthanized following approved protocols, and leg bones were carefully cleaned of muscle tissue. Bone marrow cells were then flushed out using a 25-gauge needle and a 5-ml syringe filled with ice-cold 1× PBS supplemented with 1% FBS and 2 mM EDTA. The collected bone marrow cells were centrifuged at 1,500 rpm at 4 ℃, the supernatant was removed, and the pellet was resuspended in the same cold buffer. Cells were then labeled with PE-conjugated anti-Ly6G (BioLegend), incubated with anti-PE microbeads (Miltenyi Biotec), and isolated using MACS columns (Miltenyi Biotec).

Transwell assay for migration

Neutrophil migration was assessed using an 8 μm-pore Transwell system (Beyotime Biotechnology). The bottom chamber was filled with Advanced RPMI containing 20 ng/mL CXCL1 (BioLegend). Neutrophils were pre-incubated with cLipo-DC (2-DG: 0.80 mg/mL, CQ: 0.20 mg/mL) in Advanced RPMI and seeded in the upper chamber (0.1×10^6 cells). Cells were incubated at 37 °C with 5% CO_2 for 8 hours. Migrated cells attached to the lower surface of the membrane were fixed with 4% formaldehyde at room temperature for 30 minutes, stained with 1% crystal violet solution (Beyotime, China), and subsequently imaged and quantified.

Neutrophil Depletion and Inhibition of Migration in Mice

To deplete neutrophils, mice were administered a 400 μg intraperitoneal injection of anti-mouse Ly6G antibody (108454, BioLegend) or an IgG2b isotype control (400675, BioLegend) 24 hours prior to cLipo-DC administration. Thereafter, additional 200 μg antibody injections were given weekly. Mice were euthanized after three weeks of cLipo-DC treatment. To inhibit neutrophil migration, Navarixin (Selleck, 3 mg/kg) was suspended in 0.4% methylcellulose and administered orally by gavage 24 hours before cLipo-DC administration. Additionally, a second dose of Navarixin was given concurrently with cLipo-DC administration. Control animals received an equivalent volume of 0.4% methylcellulose (10 mg/kg).

Assessments of ROS

Tissue ROS levels were detected by fluorescent probes on frozen slides using Dihydroethidium (S0063, Beyotime). Subsequently, fluorescence was examined using a confocal fluorescence microscope (LSM800, ZEISS).

Hemolysis of cLipo-DC

To measure the hemolysis of cLipo-DC, 2% sheep red blood cells (CTCC-001-0802, Meisen CTCC) were centrifuged at 371g for 10 minutes and resuspended in PBS, repeating this three times. Then, 0.25 mL of cLipo-DC at various concentrations (2-DG: 2, 4, 8 mg/mL; CQ: 0.5, 1, 2 mg/mL) was incubated with 0.5 mL of red blood cells at 37°C for 1 hour.

Triton X-100 (0.1%) and PBS served as positive and negative controls, respectively. After incubation, samples were centrifuged at 371g for 10 minutes. Absorbance of the supernatant at 576 nm was measured using a microplate reader (Nano Quant, Tecan). Hemolysis was calculated as: Hemolysis (%) = (A sample – A negative) / (A positive – A negative) × 100%.

Histological analyses

Immunofluorescence, immunohistochemistry and H&E staining were performed in paraffin-embedded mouse tissue sections. Slides were scanned using a confocal fluorescence microscope (LSM800, ZEISS) or Olympus IX71 fluorescence Microscope. The stain signal was quantified by monitoring the average number of positively stained cells or the mean fluorescence intensity from four randomly fields using ImageJ software.

Table S1. Characteristics of different liposomal formulations.

Formulation	LC (%)		EE (%)	
	2DG	CQ	2DG	CQ
cLipo-D	20.95±1.02	/	54.13±1.12	/
cLipo-C	/	10.60±1.07	/	45.93±4.67
cLipo-DC	18.45±0.48	5.04±0.38	48.39±1.26	35.31±2.65

Figure S1

cFLFLFK

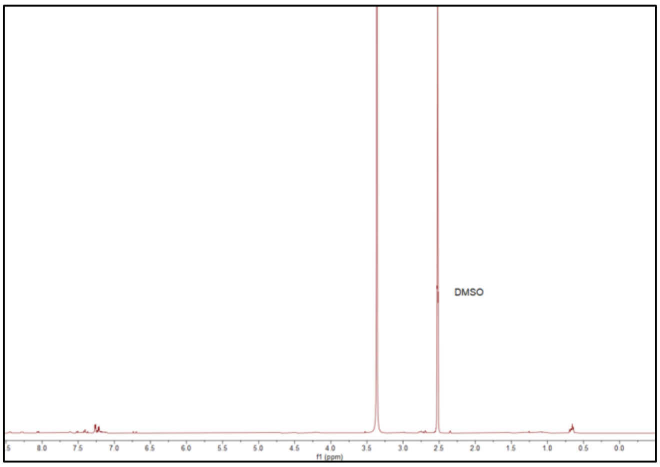

DSPE-PEG$_{2000}$-cFLFLFK

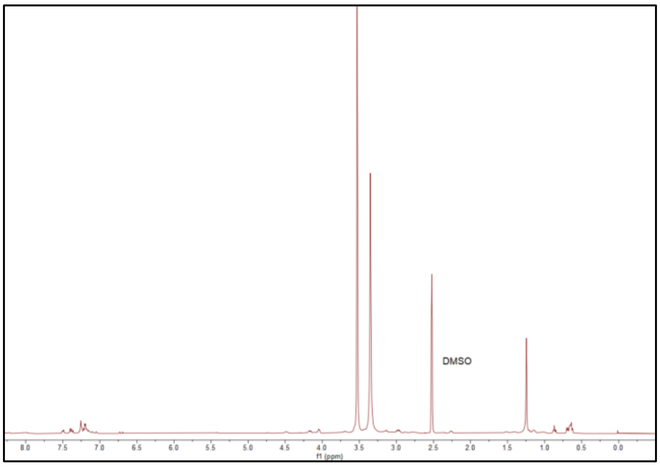

Figure S1. ^{1}H-NMR spectrum of DSPE-PEG$_{2000}$-cFLFLFK and cFLFLFK peptide.

Figure S2

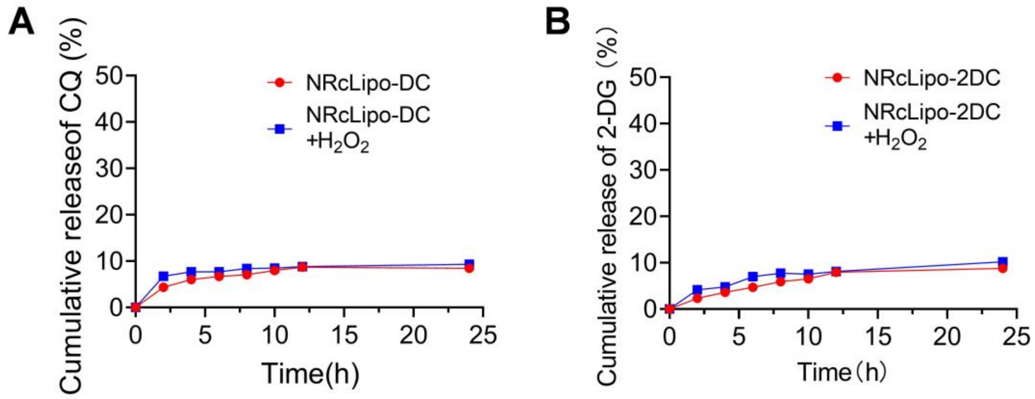

Figure S2. In vitro (A) CQ and (B) 2-DG release profiles at PBS with or without H_2O_2.

Figure S3

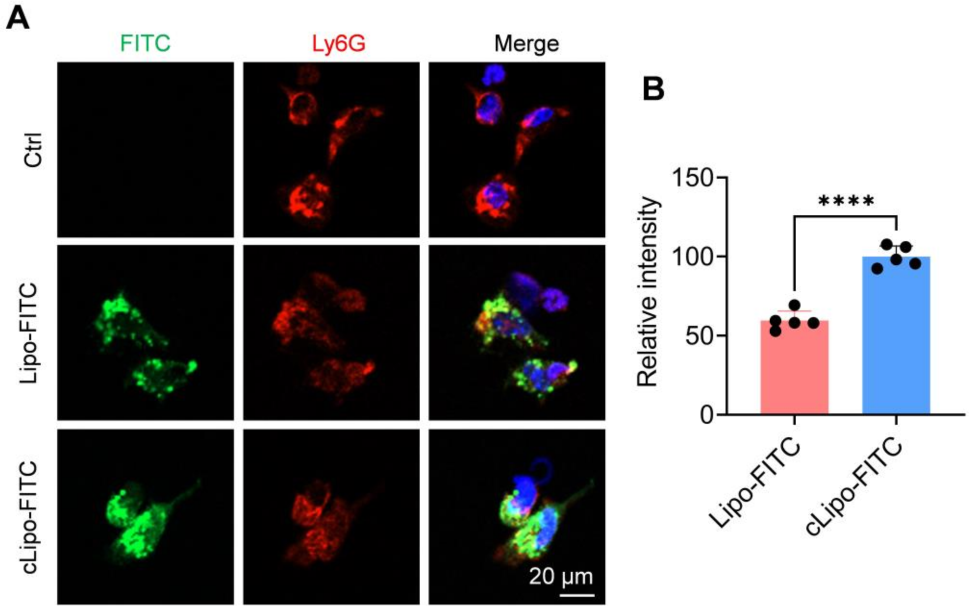

Figure S3. (A) Representative confocal images showing the internalization of liposomes (FITC) by neutrophils (Ly6G). (B) Statistical results of fluorescence intensity of liposomes.

Figure S4

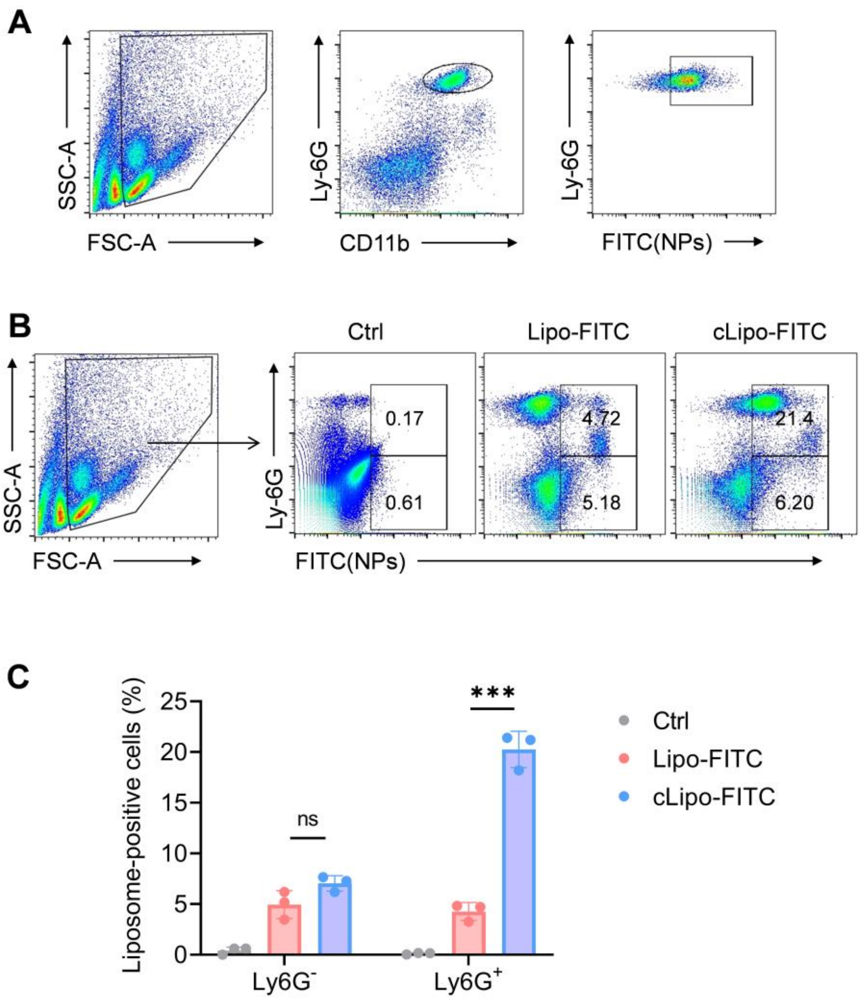

Figure S4. (A) Gating strategy of liposome-positive neutrophils. (B) Flow cytometry analysis of the percentage of liposome-positive cells in Ly6G^{+} and Ly6G^{-} cells in peritoneal fluid.

Figure S5

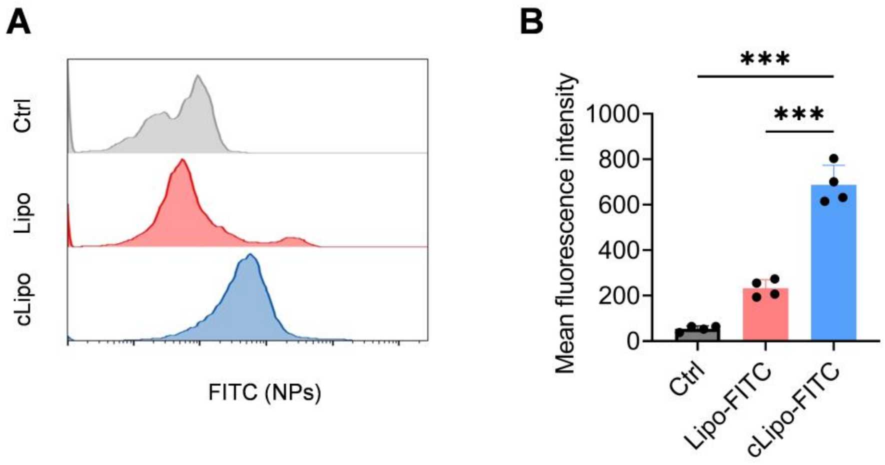

Figure S5. Flow cytometry analysis of the mean fluorescence intensity of FITC-loaded liposomes within the neutrophils in peritoneal fluid 2 hours after intraperitoneal injection. N = 4 per group.

Figure S6

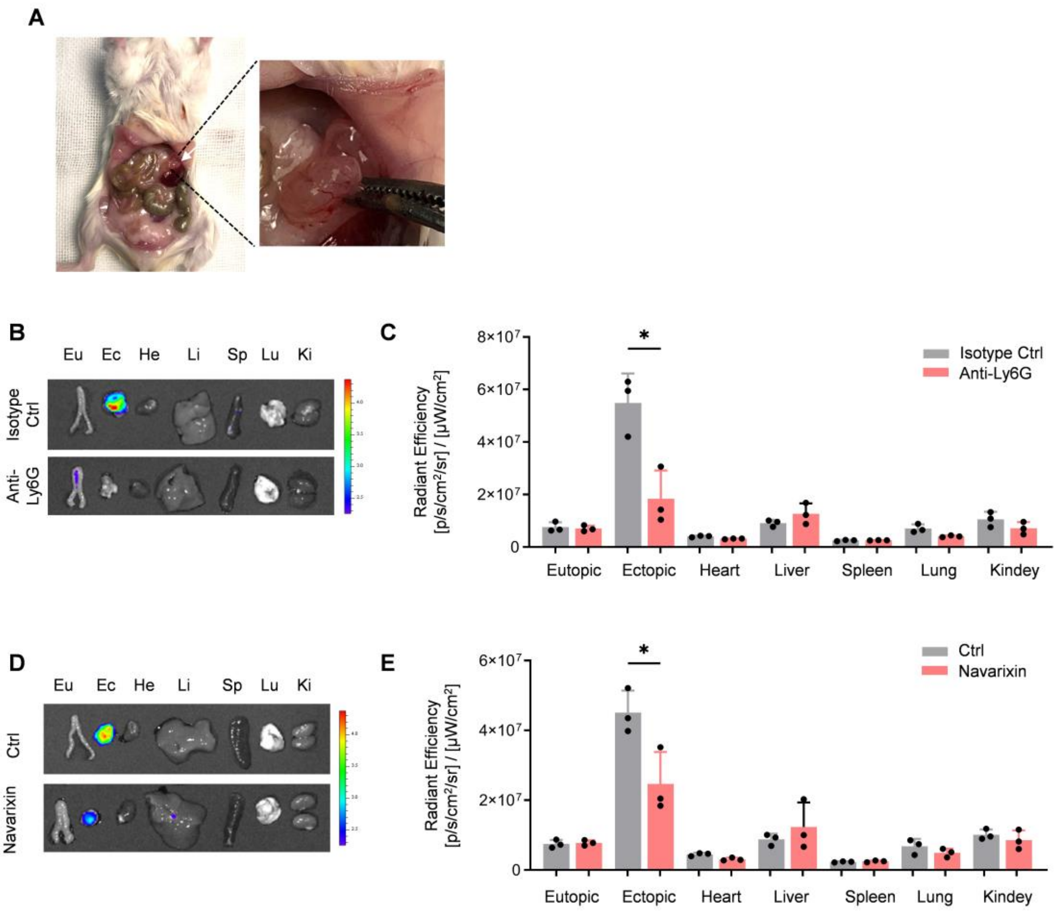

Figure S6. (A) Anatomical diagram of ectopic lesions. (B, D) Ex vivo fluorescence images and (C, E) corresponding quantification of average fluorescence intensities of eutopic endometrium, ectopic lesion and major organs collected 16 h post liposomes injection by intraperitoneal. N = 3 per group.

Figure S7

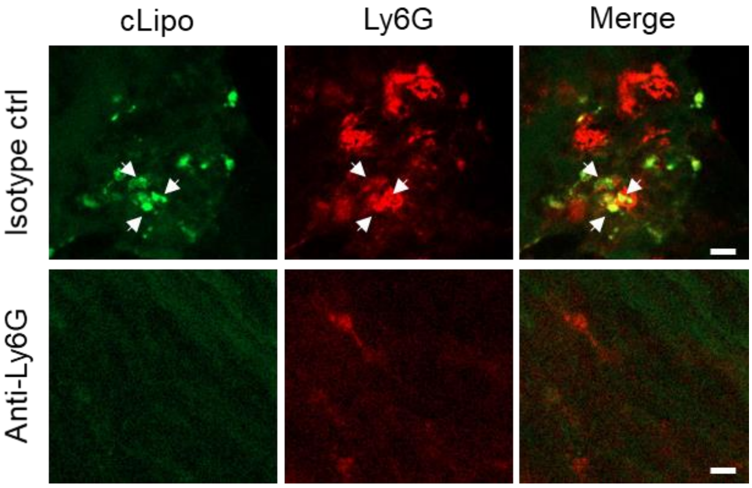

Figure S7. The immunofluorescence staining confocal images show liposomes (FITC) and neutrophils (Ly6G). Scale bar: 10 μm.

Figure S8

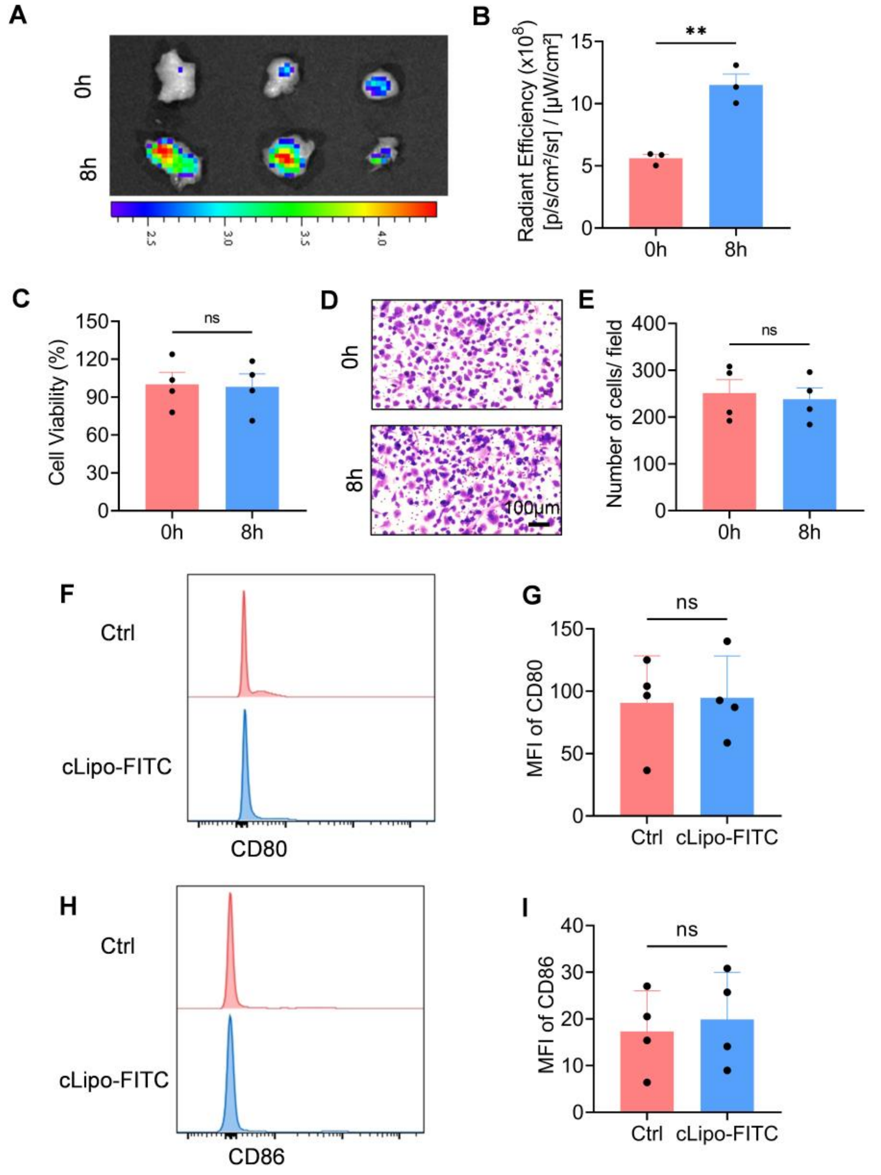

Figure S8. (A) Ex vivo fluorescence images and (B) corresponding quantification of average fluorescence intensities of ectopic lesion. N = 3 per group. (C) Relative cell viability of mouse neutrophils treated with cLipo-DC for 8 h. N = 4 per group. (D-E) Transwell assay to assess the migration of mouse neutrophils treated with cLipo-DC for 8 hours. N = 4 per group. (F-I) Flow cytometry analysis of the mean fluorescence intensity of CD80 and CD86 on neutrophils in peritoneal fluid, 2 hours after intraperitoneal injection of cLipo-DC. N = 4 per group.

Figure S9

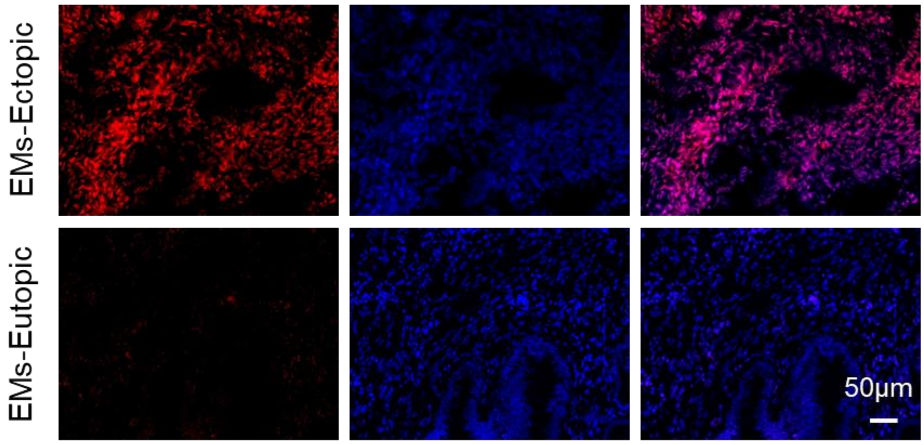

Figure S9. Fluorescent images of ROS stained by DHE (red) and nucleus (DAPI, blue) in the eutopic and ectopic endometrium of mice. Scale bar: 50 μm.

Figure S10

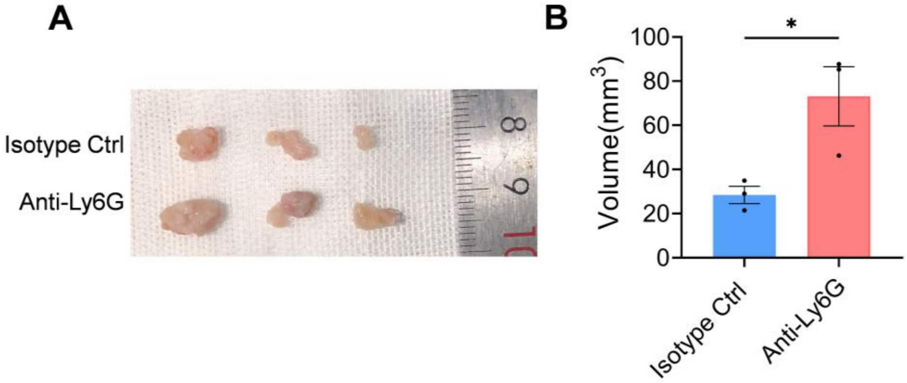

Figure S10. (A) Images and (B) total volume of endometriotic lesions in mice following different treatments. N = 3 per group.

Figure S11

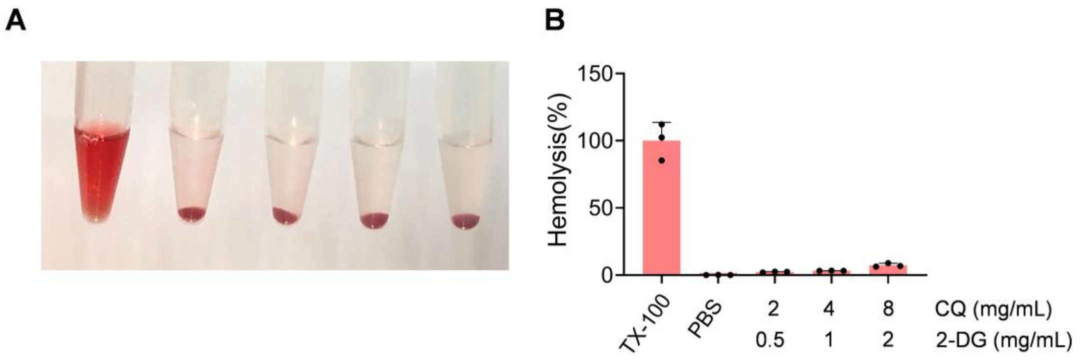

Figure S11. (A) Representative images of sheep red blood cells and (B) hemolysis assay in the presence of 1% Triton X-100 (TX-100), PBS, or various concentrations of cLipo-DC, respectively. Data are shown as the mean ± SD. N = 3.

Figure S12

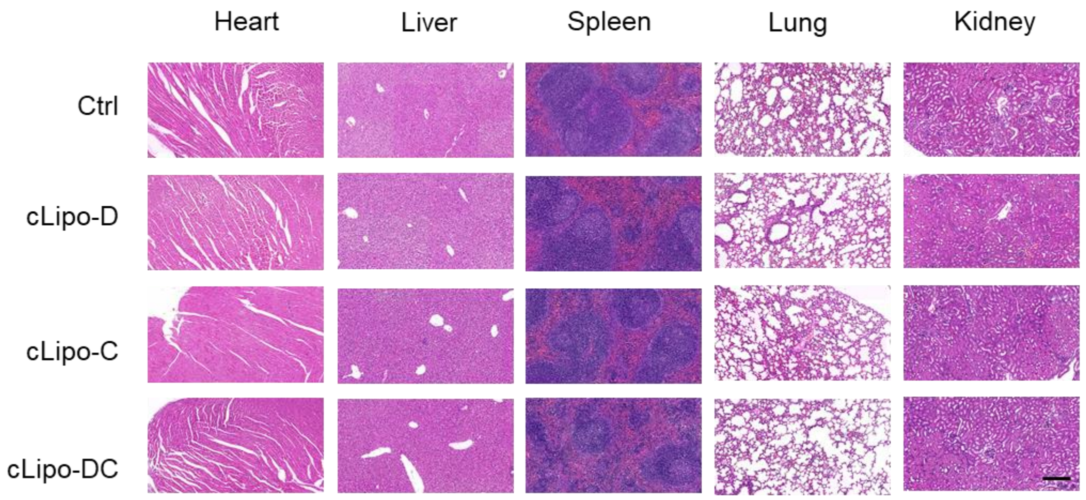

Figure S12. Hematoxylin and eosin (H&E) staining of major organs (heart, liver, spleen, lung, and kidney) of mice treated with different formulations. Scale bar: 200 μm.
